# Temporal persistence of after-effects in the n-1 replication task

**DOI:** 10.3758/s13414-020-02073-4

**Published:** 2020-06-16

**Authors:** Oliver Simon Sack, Christine Sutter

**Affiliations:** 1 Institute of Traffic and Engineering Psychology, German Police University, Zum Roten Berge 18-24, 48165 Münster, Germany; 2 Work and Cognitive Psychology, RWTH Aachen University, Jägerstraße 17-19, 52056 Aachen, Germany

**Keywords:** Theory of event coding, Stimulus-response binding, Cross talk, Human information processing, Tool use

## Abstract

In line with the theory of event coding, many studies on tool use show that perceived visual and haptic information interacts with action execution. In two experiments, we investigated the temporal persistence of after-effects within an event file, and after-effects in temporally overlapping event files with the *n-1* replication task. Each trial consisted of two phases: In phase 1, participants moved a cursor with a pen on a covered tablet while a gain varied the relation between hand and cursor amplitude (Experiment 1). In phase 2, participants replicated the hand amplitude of phase 1 of the previous trial without visual feedback. Any systematic over- and undershoot would be indicative for after-effects. When the cursor amplitude varied and the hand amplitude remained constant, we did not find any after-effects but adjustment of the internal model. For varying hand amplitudes, after-effects appeared in terms of a *contrast* and *assimilation effect* between temporally overlapping event files and within an event file, respectively. In Experiment 2, we confirmed that the observed pattern of over- and undershoots fully reflect assimilation/contrast due to perception-action interaction. The findings extend the current view on the temporal stability of short-term binding in sensorimotor transformation tasks: In the *n-1* replication task, after-effects appeared only in trials with varying hand amplitudes. We replicated the *contrast effect* and *assimilation effect*, and the *assimilation effect* persisted for up to approximately 20 s.

## Introduction

**Fig. 1:**
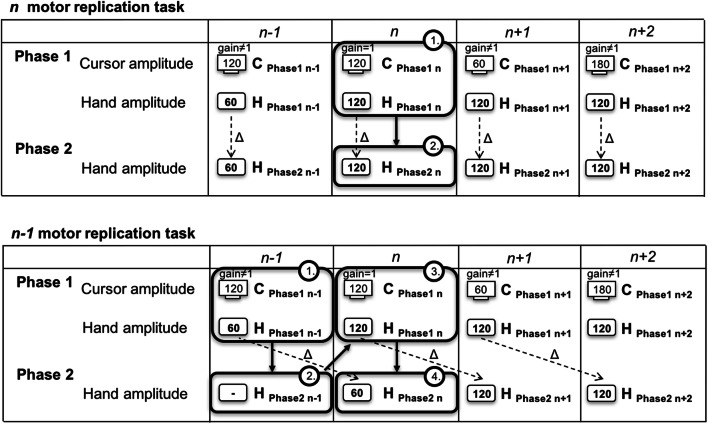
*N* replication task (**top**) and *n-1* replication task (**bottom**). Phase 1 of trial *n:* Participants move the cursor with a pen on a covered tablet while a gain varies the relation between hand and cursor amplitude. Phase 2 of trial *n*: Participants replicate the hand amplitude of phase 1 of trial *n* (top, *dashed arrow*) or of the previous trial *n-1* (bottom, *dashed arrow*) without visual feedback. Solid ovals indicate the temporal order

In many situations of modern tool use, users face sensorimotor transformations. For example, users perform shorter amplitudes with a computer mouse than the cursor moves on a display. Many studies investigated the effects of such sensorimotor transformations on manual action control and found after-effects in motor actions (for an overview, see Sutter, Sülzenbrück, Rieger, & Müsseler, 2013). The present study further investigates the interaction between perception and action within a distinct event file and between temporally overlapping event files, their temporal persistence, and their effects on motor responses. From a cognitive point of view and in line with the theory of event coding (TEC; Hommel, Müsseler, Aschersleben, & Prinz, 2001), findings of perception-action interaction can be attributed to interactions between the late stage of sensory information processing and the early stage of motor response. The TEC proposes that perceived visual and haptic information and action are represented in a common temporary, distinct event file, and therefore perception and action are likely to interact with each other (= cross talk). The theory provides a framework for the cognitive representation of perception and action in a common representational domain – a short-term binding between the late stage of sensory information processing and the early stage of motor response. This concept of an event-related binding goes back to the ideomotor principle (Greenwald, 1970; James, 1890). The ideomotor principle postulates that the sensory action effect of an intended action is predefined by anticipating the sensory consequences of that action. In addition, the common coding approach (Prinz, 1997) claims that perception and action share a common distal representational domain. In this domain, feature codes of perception as well as feature codes of action are similar in kind. The TEC (Hommel et al., 2001) is an extension of both approaches. The core concept of TEC is the event code. An event code can represent either a perceptual or a planned event. The event code itself comprises an assembly of distal feature codes that are the attributes of a perceived or a (to-be)-produced event. During the last years and based on TEC, the idea of *event file* (“transient bindings between stimulus and/or action features,” Hommel, 2004, p. 494) gained interest to explain various findings of interactions between perception and action in discrete or continuous stimulus-response tasks (for an overview, see Hommel, 2011; Sutter et al., 2013). So far, most studies demonstrated those transient bindings in discrete motor actions that required an immediate response to stimuli. These studies found after-effects in terms of short-term biases (e.g., in a Simon task: Stürmer, Leuthold, Soetens, Schröter, & Sommer, 2002; Sutter & Ladwig, 2012; in motor replication tasks: Ladwig, Sutter, & Müsseler, 2012, 2013; Perrotin & d’Alessandro, 2016; Sack & Sutter, 2017; Wendker, Sack, & Sutter, 2014). For instance, Ladwig et al. (2012, 2013, Exp. 1) introduced a *n* replication task (Fig. 1, top). Each trial consisted of two phases: In phase 1, participants moved a cursor on a display from one bar to another bar with the pen on a covered tablet. The relation between cursor and hand amplitude varied between trials. The cursor amplitude was longer, equal to or shorter than the hand amplitude. In phase 2, participants replicated the formerly performed hand amplitude without any visual feedback. In the framework of TEC (Hommel et al., 2001), each event file consisted of sensory codes (visual and haptic information of phase 1) and a motor code (phase 2). As the dependent variable, the mean deviation between hand amplitude in phase 2 and hand amplitude in phase 1 was calculated (Fig. 1, top, dashed arrow). When in phase 1 cursor and hand amplitude corresponded, replications were precise. Effects of sensorimotor transformation appeared in trials in which cursor and hand amplitude did not correspond in phase 1. When in phase 1 the cursor amplitude was shorter than the hand amplitude, participants undershot. On the contrary, when in phase 1 the cursor amplitude was longer than the hand amplitude, participants overshot. The systematic deviations between hand amplitudes in phase 1 and phase 2 were interpreted as after-effects, since visual and haptic information (phase 1) and motor response (phase 2) assimilated.

Moreover, Schubö, Aschersleben, and Prinz (2001) demonstrated effects of short-term bindings in a continuous task that required a delayed motor response. They introduced a serial overlapping response task. In trial *n,* participants replicated the sinusoidal-like stimulus motion that was presented on the screen in trial *n-1*. Simultaneously, participants encoded a new stimulus that had to be replicated in the subsequent trial *n+1*. In the framework of TEC (Hommel et al., 2001), each event file consisted of sensory codes (visual information in trial *n-1*) and a motor code (in trial *n*), and temporally overlapped with another event file (visual information in trial *n*, and motor response in trial *n+1*). The findings showed a contrast effect: When the simultaneously presented visual amplitude was shorter than the hand amplitude, the replication was longer than required. When the simultaneously presented visual amplitude was longer than the hand amplitude, the replication was shorter than required. The contrast effect even appeared with a temporal onset asynchrony between stimulus motion and hand movement (−500 ms, +500 ms). The authors explained that on each trial stimulus motion and hand movement are not assigned to each other but the two independent event files (termed “S-R assignments” by Schubö et al., 2001) temporally overlap in each trial. Due to code modification, activation shifts from overlapping features between event files (e.g., sinusoidal-like motion pattern) toward distinguishing features (e.g., amplitude length). This shift toward distinctions between event files accounts for the contrast effect. The studies showed that transient bindings between sensory and motor codes occur in discrete and continuous tasks. The binding in discrete and temporally non-overlapping event files resulted in an assimilation effect. In temporally overlapping event files, the binding provoked a contrast effect. Schubö et al. (2001) further demonstrated a temporal persistence of the binding up to 500 ms.

The present paper further investigates the temporal persistence of transient bindings in temporally overlapping and non-overlapping event files, and their effects on motor responses (i.e., after-effects). So far, studies focused on transient bindings in either temporally overlapping or non-overlapping event files (e.g., Ladwig et al., 2012, 2013; Schubö et al., 2001). To further investigate the flexibility of short-term bindings under changing task conditions, the present study will therefore merge temporally non-overlapping event files (Ladwig et al., 2012, 2013, Exp.1) and temporally overlapping event files (Schubö et al., 2001) in the experimental design. The experimental design is based on the framework of TEC, and according to this, we will investigate assumptions of the linkage between the late stage of sensory information processing and the early stage of motor response. However, to extend the view, we will refer to alternative approaches, such as sensorimotor adaptation and optimal multisensory integration.

We developed the *n-1* replication task (Fig. 1, bottom) with the same features as described above for the *n* replication task (cf., Ladwig et al., 2012, 2013, Exp.1), except that in phase 2, participants replicate the hand amplitude of phase 1 of the previous trial. Again, each trial consists of two phases. In phase 1 of trial *n,* participants move a pen on a covered digitizer tablet while a gain varies the relation between hand amplitude and cursor amplitude (either varying hand amplitude or varying cursor amplitude). Participants are instructed to monitor the hand amplitude in phase 1 very carefully for the later replication. In phase 2 of trial *n*, participants replicate the hand amplitude of phase 1 of the previous trial *n-1* without any visual feedback. Following the same rationale for the definition of event files as described earlier (i.e., Hommel et al., 2001; Ladwig et al., 2012, 2013; Schubö et al., 2001), we assume that each event file consists of sensory codes (visual and haptic information of phase 1 of trial *n-1*) and a motor code (phase 2 of trial *n*). Thus, overlapping feature codes (e.g., same shape and direction of hand and cursor motion in phase 1) as well as not overlapping feature codes (e.g., different length of cursor and hand amplitude) are activated in phase 1 of trial *n-1*. Activated features are integrated in the event file. Sensory and motor codes of an event file are likely to interact with each other, so that sensory information affects motor responses. The degree of this interaction between perception and action are calculated by the mean deviation between hand amplitude in phase 2 of trial *n* and hand amplitude in phase 1 of trial *n-1* (Fig. 1, bottom, dashed arrow).

The overlap of event files, and the distinct event files are implemented as follows. In the experiments, we introduce two different sequences. Each sequence consists of two trials, and contains two event files: Event file 1 consists of sensory codes (visual and haptic information in trial *n-1*) and a motor code (in trial *n*), and temporally overlaps with event file 2 (visual and haptic information in trial *n*, and motor response in trial *n+1*). A sequence is further defined by the presentation order of the gain=1 trial and gain≠1 trial. The number of the sequence represents the presentation position of the gain=1 trial. The presentation order of gain=1 trials and gain≠1 trials for each sequence is now described in detail.

In sequence 1 (gain=1/gain≠1), the gain=1 trial is presented first. That means, a gain=1 in trial *n-1* (relation between cursor and hand amplitude is 120:120) precedes a gain≠1 in trial *n* (e.g., 60:120). In sequence 2 (gain≠1/gain=1), the gain=1 trial is presented second: The gain=1 in trial *n* (120:120) follows a gain≠1 in trial *n-1* (e.g., 120:60). Sequence 1 is of special interest to investigate after-effects that might emerge from the temporal overlap of event files. In this sequence, visual and haptic information does correspond in phase 1 of trial *n-1* and does not correspond in phase 1 of trial *n*. We assume that sensory and motor codes of event file 1 (gain=1) do not interact. However, event file 1 is affected by the temporally overlapping event file 2, in which sensory information does not correspond (gain≠1). The sensory codes (phase 1 of trial *n*) and motor codes (phase 2 of trial *n*) are not assigned to each other, but temporally overlap. With respect to selective code modification account (Schubö, Prinz, & Aschersleben, 2004), activations of event codes should drift in opposite direction in order to separate events. As a result, sensory codes (visual and haptic information of phase 1 of trial *n*) might induce a contrast-like effect on the *n-1* replication in phase 2 of trial *n* (= *contrast effect*, Hypothesis 1). We expect that when in phase 1 of trial *n* the cursor amplitude is longer than the hand amplitude, participants undershoot. When in phase 1 of trial *n* the cursor amplitude is shorter than the hand amplitude, participants overshoot.

However, any observed contrast effect could also be caused by adaptation or adjustments of the internal model (Wolpert & Flanagan, 2001). According to Welch (1978), the term adaptation describes both the process to adjust to a discrepancy of not corresponding perception and action as well as the end state of this adjustment process. Considering the present *n-1* replication task, any action performed in phase 1 will generate an internal model of the cursor-hand relation. Trial-by-trial, participants will adjust the internal model to the present cursor-hand relation in phase 1. In trials with gain=1, the internal model will be exact since the relation between hand and cursor amplitude corresponds. In trials with gain≠1, there is some uncertainty about the relation between hand and cursor amplitude. Since the gain varies from trial to trial, participants will not be able to adjust to a gain fully, but they might implicitly learn the range of sensorimotor gains. When in phase 2 participants replicate the hand amplitude of phase 1 of the previous trial, they will rely (at least partially) on the adjusted forward model to which they adapted in phase 1 of the current trial.

For adaptation to gain changes, Rieger et al. (2005) asked participants to perform a drawing task with continuous up- and downward strokes. A gain varied the relation between cursor and hand amplitude, and changed every six strokes. In experimental conditions, the cursor amplitude was either shorter or longer than the hand amplitude (gain≠1). In the baseline condition, cursor and hand amplitude corresponded (gain=1). It was found that the previous forward model was still in effect in the first trial after a gain change, and was fully adjusted to the current gain within five strokes. For the *n* replication task, Wendker et al. (2014) demonstrated reduced after-effects in gain repetition-trials compared to gain change-trials. This benefit from gain repetitions expresses the adjustment of the forward model (Wolpert & Flanagan, 2001).

In the following, we define adaptation in sensorimotor transformations as an adjustment of the internal model (i.e., in particular, the forward model that modulates the causal relation between motor action and sensory consequences; Wolpert, Doya, & Kawato, 2003) to a changing environment. Such an adjustment can either appear in situations of a gain change between trials (e.g., changing from gain≠1 to gain=1, cf. Rieger et al., 2005; Wendker et al., 2014) or as refinement within a trial. Concerning our experiment, we assume that sensorimotor adaptation produces an internal model of the cursor-hand relation after even one trial of gain≠1. In sequence 1, we do not expect any adaptation in trials with gain=1, and effects of sensorimotor adaption in trials with gain≠1. Consequently, this would lead to the same contrast effect as hypothesized above (cf., Hypothesis 1).

With sequence 2 (gain=1 trial second), we investigate perception-action interaction and its temporal persistence within a distinct event file. In this sequence, perceived visual and haptic information does not correspond in phase 1 of trial *n-1*, and does correspond in phase 1 of trial *n*. Since perception and action concerning an event share a common representational domain (Hommel et al., 2001), it is likely that sensory information from different modalities interact and affect subsequent motor responses. We assume that sensory and motor codes of event file 1 (gain≠1) interact. However, event file 1 will not be affected by the temporally overlapping event file 2, since sensory codes in event file 2 will not cause any conflict (gain=1). Comparable to Ladwig et al. (2012, 2013, Exp.1), any systematic deviation between replicated hand amplitude in phase 2 of trial *n* and hand amplitude in phase 1 of trial *n-1* would be indicative for assimilation of visual information from the previous cursor amplitude in trial *n-1* in replications of trial *n* (= *assimilation effect*; Hypothesis 2). When in phase 1 of trial *n-1* the cursor amplitude is longer than the hand amplitude, participants overshoot. And the other way around, when in phase 1 of trial *n-1* the cursor amplitude is shorter than the hand amplitude, participants undershoot.

Whereas TEC (Hommel et al., 2001) provides a framework for the linkage between the late stage of sensory information processing and the early stage of motor response, the principle of maximum-likelihood estimation (MLE; Ernst & Banks, 2002) considers bottom-up multisensory information processing on the very early stage of sensory information processing. If the nervous system receives sensory information from more than one source, the (redundant) sensory information is combined in a statistically optimal fashion: For any sensory information, stimulus properties are estimated for each single modality. Noise corrupts the estimates, as it induces variance in the signal. For instance, high visual contrast enhances visual target detection, and variance in responses is low. Downscaling visual contrast makes it more and more difficult to detect the visual target, and variance in responses increases. Minimizing the variance in the combined percept leads to optimal integration of multisensory information. Each single modality estimate contributes to the combined percept with its reciprocal variance. Consequently, the variance for the combined percept is lower than the variance for each single modality estimate. For instance, when increasing the variance in the haptic modality, the visual modality dominates in perception (e.g., Ernst & Banks, 2002; Reuschel et al., 2010; Takahashi, Diedrichsen, & Watt, 2009) and action control (e.g., Knoblich & Kircher, 2004; Ladwig et al., 2013 Exp. 2; Massen & Prinz, 2007; Sülzenbrück & Heuer, 2009; Sutter & Ladwig, 2012; Sutter & Müsseler, 2010). For haptic dominance, the opposite is the case in perception (e.g., Ernst & Banks, 2002) and action control (e.g., Sutter & Ladwig, 2012; Sutter et al., 2013).

Computational and cognitive approaches (Ernst & Banks, 2002; Hommel et al., 2001) are consistent with and complementary to each other since MLE and TEC agree on the idea of a common representational domain that integrates sensory information from different modalities in the earlier stage of information processing. Concerning the temporal persistence of short-term binding in our experimental task, both approaches (Ernst & Banks, 2002; Hommel et al., 2001) would link the sensory integration of visual and haptic information to phase 1 of each trial. The TEC furthermore assumes a short-term binding between perception and action. If in sequence 1, short-term binding persists over time (in this case from phase 1 in trial *n-1* to phase 2 in trial *n*), after-effects in terms of an assimilation effect should reflect the visual dominance in perception-action interaction.

## Experiment 1

### Method

#### Participants

Twelve students (nine female) of the RWTH Aachen University, aged from 19 to 25 years (M= 21.8; SD= 2.4), volunteered. Before taking part in the study, all participants gave their informed consent. All but two participants were right-handed. Participants had normal or corrected-to-normal vision, and received credit for a course or were rewarded for their efforts with 7.50 €.

#### Apparatus, task, and stimuli

**Fig. 2:**
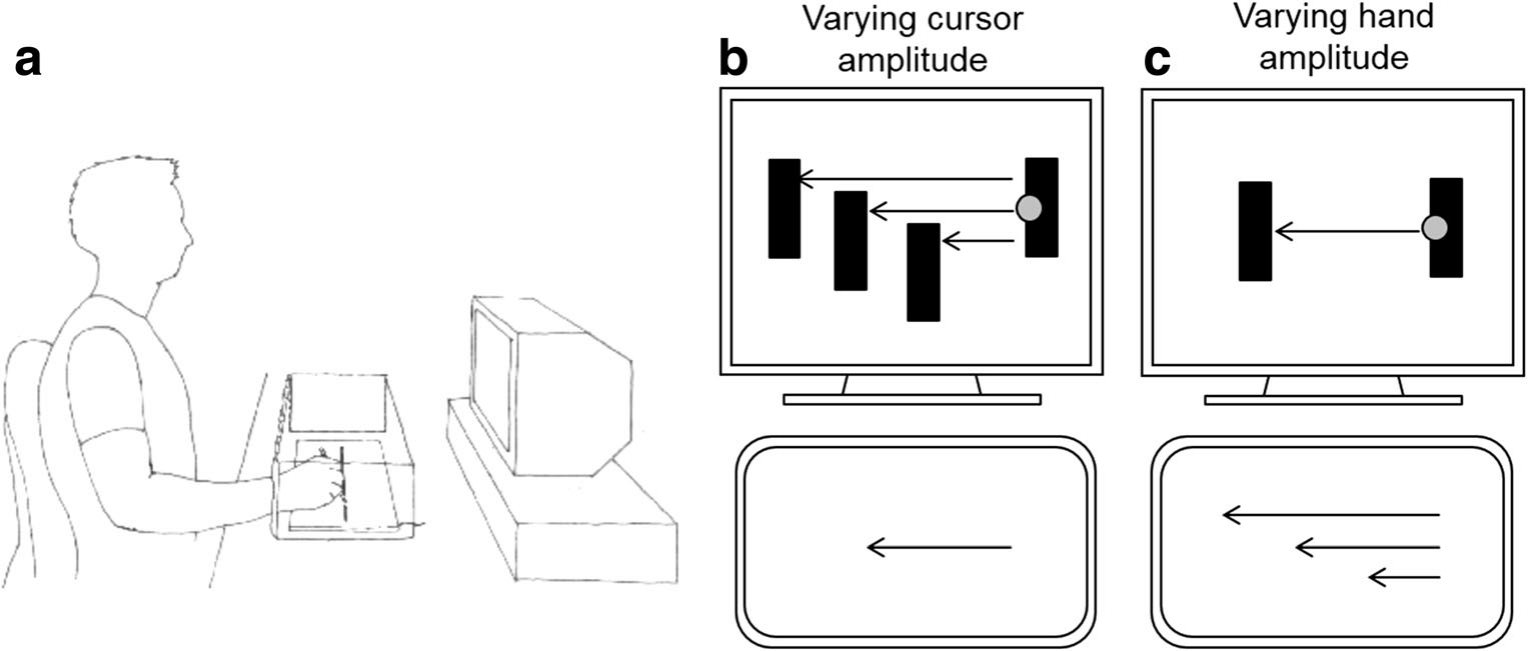
Experimental setup (**a**), varying cursor amplitude (**b**), and varying hand amplitude (**c**) in phase 1

The experiment was carried out in a dimly lit room and controlled by an Apple Macintosh computer running Matlab software with the Psychophysics Toolbox extension (Kleiner, Brainard, & Pelli, 2007). Figure 2a depicts the experimental setup. Participants sat in front of a DIN-A3 digitizer tablet (WACOM Intuos2, 100-Hz sampling rate) and a 22-in. color CRT display (Iiyama HM204DT Vision Master Pro514, 1,024 × 768 pixel, 100-Hz refresh rate). They guided the pen (WACOM Intuos2 Grip Pen) on the tablet with their dominant hand while the non-dominant hand rested in their lap. Participants pressed the button on the pen three times per trial: to start a trial (first click), and to indicate the pen’s final positions at the end of phase 1 (second click) and phase 2 (third click). A foam rubber with a cut-out groove (width and length of the groove 4 and 450 mm) was mounted onto the digitizer tablet’s active area. To perform horizontal strokes on the tablet, participants moved the tip of the pen within the groove. A wooden occluder with a curtain screened the digitizer tablet and the moving hand from view. The instructions, the stimuli, and the cursor movement were presented on the display (viewing distance approximately 600 mm between participant and display).

The following data were recorded in a data file (100-Hz sampling rate) for each participant: cursor and hand amplitude in phase 1 (mm), hand amplitude in phase 2 (mm), mean deviation between the hand amplitude in phase 2 of trial *n* and the hand amplitude in phase 1 of trial *n-1* (mm), and errors (i.e., when the second button click occurred while the cursor was outside the target bar, and/or when the replicated hand amplitude was shorter than or equal to 10 mm).

In addition to that, the experimenter sat next to the participant and monitored the velocity and direction of the participant’s hand movement displayed on a second display. The distance-time graph was also recorded for each participant and each trial (100-Hz sampling rate). If a participant erroneously changed the movement direction within a phase (e.g., after overshooting the target bar in phase 1) this error was recorded manually on an error sheet, and later on manually recorded in data files.

Treatment of error trials: All error trials in the data were eliminated, and not further analyzed. Trials were considered as erroneous when the trajectories of hand movement in phase 1 and phase 2 were non-continuous (with velocity = 0 within movement), when the initial movement direction changed, when the initial hand movement overshot the target bar, when the second button click occurred while the cursor was outside the target bar (recorded by software), and/or when the replicated hand amplitude was shorter than or equal to 10 mm (recorded by software). Additionally, each trial following an error trial was omitted as well.

As depicted in Fig. 2 for trials with varying cursor amplitudes (Fig. 2b) and trials with varying hand amplitudes (Fig. 2c), phase 1of each trial started with the presentation of two black bars (rectangles of 2 × 8 mm each) and a gray circular cursor (diameter 4 mm). The cursor was positioned onto the right (start) bar, and the task in phase 1 was to move the cursor from the start bar to the opposite (target) bar as accurately as possible by moving the pen leftward along the groove on the digitizer tablet. When the cursor had reached the target bar, phase 2 started. The screen turned blank, and participants had to reverse the movement direction and to move the pen rightward without any visual feedback. The task in phase 2 required replicating the hand amplitude of phase 1 of the previous trial as accurately as possible. For half of participants the start position was on the right side. For the other half, the start position was on the left side (not depicted in Fig. 2). In phase 1, three different gain factors varied the relation between cursor and hand amplitude. Figure 2b depicts the task for varying cursor amplitudes. The applied gain factors (1.5:1, 1:1, 0.5:1) resulted in cursor amplitudes of 180, 120, or 60 mm, so that the cursor amplitude was longer, equal to, or shorter than the constant hand amplitude (120 mm). When in phase 1 of the previous trial the hand amplitude was 120 mm, phase 2 required moving the pen by 120 mm.

Figure 2c depicts the task for varying hand amplitudes. The applied gain factors (1:1.5, 1:1, 1:0.5) resulted in hand amplitudes of 180, 120, or 60 mm, so that the hand amplitude was longer, equal to, or shorter than the constant cursor amplitude (120 mm). When in phase 1 of the previous trial the hand amplitude varied (180, 120, or 60 mm), phase 2 required moving the pen to the same amount (180, 120, or 60 mm).

### Procedure and design

The experiment consisted of a practice block and an experimental block. Before the practice block and the experiment started, participants were instructed to move as accurately as possible and to produce continuous and smooth forth and back movements with the pen without interrupting. They were further instructed to monitor the hand amplitude carefully since they had to replicate this hand amplitude as accurately as possible. At the beginning of each trial, the cursor, the start bar, and the target bar were presented on the screen. Participants were instructed to move the cursor from the start bar to the target bar by moving the pen on the tablet. A first click of the pen’s button unlocked the cursor, and participants moved it to the opposite target bar while receiving continuous visual feedback. When the cursor overlapped the target bar (accepted overlap 1.8–2 mm, related to the vertical centerline of the target bar), participants pressed the pen’s button a second time. Then, both bars and the cursor disappeared, and participants started the replication by reversing the movement direction with the pen. When it was thought they had replicated the required distance (i.e., hand amplitude of phase 1 of the previous trial), they finally pressed the pen’s button again to terminate the trial. The next trial started from the end position of the previous trial. Participants were randomly assigned to movement directions. The experiment lasted about 45 min.

The practice block consisted of 12 gain≠1 trials and nine gain=1 trials (in total 21 trials). The relation between cursor and hand amplitude for gain≠1 trials were 120:60, 120:180, 60:120, and 180:120 (three repetitions each), and the relation between cursor and hand amplitude for gain=1 trials was 120:120 (nine repetitions). The presentation order of gain≠1 and gain=1 trials was fixed within the practice block. The practice block started and ended with a gain=1 trial. Practice trials were presented in advance of the experimental block in order to familiarize participants with the task.

The experimental block consisted of 48 gain≠1 trials and 33 gain=1 trials (in total 81 trials). The relations between cursor and hand amplitude for gain≠1 trials were 120:60, 120:180, 60:120, and 180:120 (12 repetitions each), and the relation between cursor and hand amplitude for gain=1 trials was 120:120 (33 repetitions). The experimental block always started and ended with a gain=1 trial. Three different sequences arranged the presentation order of gain≠1 and gain=1 trials: In sequence 1 (eight repetitions per gain≠1 condition 120:60, 120:180, 60:120, and 180:120), the gain=1 trial is presented first, and precedes a gain≠1 trial. In sequence 2 (eight repetitions per gain≠1 condition), the gain=1 trial is presented second, and follows a gain≠1 trial. In sequence 3 (16 repetitions), a gain≠1 trial preceded a gain≠1 trial to avoid sequence-learning effects. The order of sequences was pseudo-random. Another sequence with gain=1/gain=1 was not presented since we did not assume any effect on short-term binding.

Since the first experimental trial (gain=1) was not assigned to any previous hand amplitude, first trials were excluded before error analysis (1.23% of all trials). In total, the error rate was 6.14%.

Concerning the determination of sample size for sufficient statistical power: Since the *n-1* replication task is an entirely new paradigm, we could not use a power analysis to estimate the necessary sample size. As such, previous studies (Ladwig et al., 2013; Sack & Sutter, 2017) using the *n* replication task guided us to determine our sample size. The aforementioned studies investigated 18–36 participants in a between-subject design with 9–12 participants per group. Results revealed large effect sizes (for the main effect gain: *η*^2^ = .89–.93; for the interaction between gain and constancy: *η*^2^ = .71–.72). In order to ensure sufficient statistical power, we collected a sample size of N = 12, and used a within-subject design with repeated measurements in each condition.

We analyzed data using a 2 (sequence: 1 (gain=1/gain≠1) vs. 2 (gain≠1/gain=1); within-subject) × 2 (constancy: varying cursor amplitude vs. varying hand amplitude; within-subject) × 2 (gain: cursor amplitude longer or shorter than hand amplitude; within-subject) analysis of variance with repeated measurement (ANOVA). We used *t*-tests to analyze whether mean deviations differed significantly from zero, and calculated Cohen’s *d* for the effect size. As dependent variable, the mean deviation (in mm; see Fig. 1, dashed arrow = ∆) between the hand amplitude in phase 2 of trial *n* and the hand amplitude in phase 1 of trial *n-1* was calculated for error-free trials. Positive values of mean deviations represent an overshoot in phase 2, negative values of mean deviations represent an undershoot in phase 2. After-effects are defined as any systematic deviation in terms of over- and undershoots (difference significant) that significantly differs from zero.

### Results and discussion

A 2 × 2 × 2 analysis of variance with repeated measurement revealed a significant main effect of the factor gain [*F*(1,11) = 5.28; *p* = .042, *η*^2^ = .32], a significant interaction of sequence and gain [*F*(1,11) = 17.35; *p* = .002, *η*^2^ = .61], a significant interaction of constancy and gain [*F*(1,11) = 12.07; *p* = .005, *η*^2^ = .52], and a significant interaction of sequence, constancy and gain [*F*(1,11) = 9.11; *p* = .012, *η*^2^ = .45]. Other main effects and interaction did not reach significance (factor sequence [*F*(1,11) = 1.59; *p* = .232, *η*^2^ = .12], factor constancy [*F*(1,11) = 0.01; *p* = .893, *η*^2^ = .002], interaction of sequence and constancy [*F*(1,11) = 0.02; *p* = .883, *η*^2^ = .002]).

**Fig. 3:**
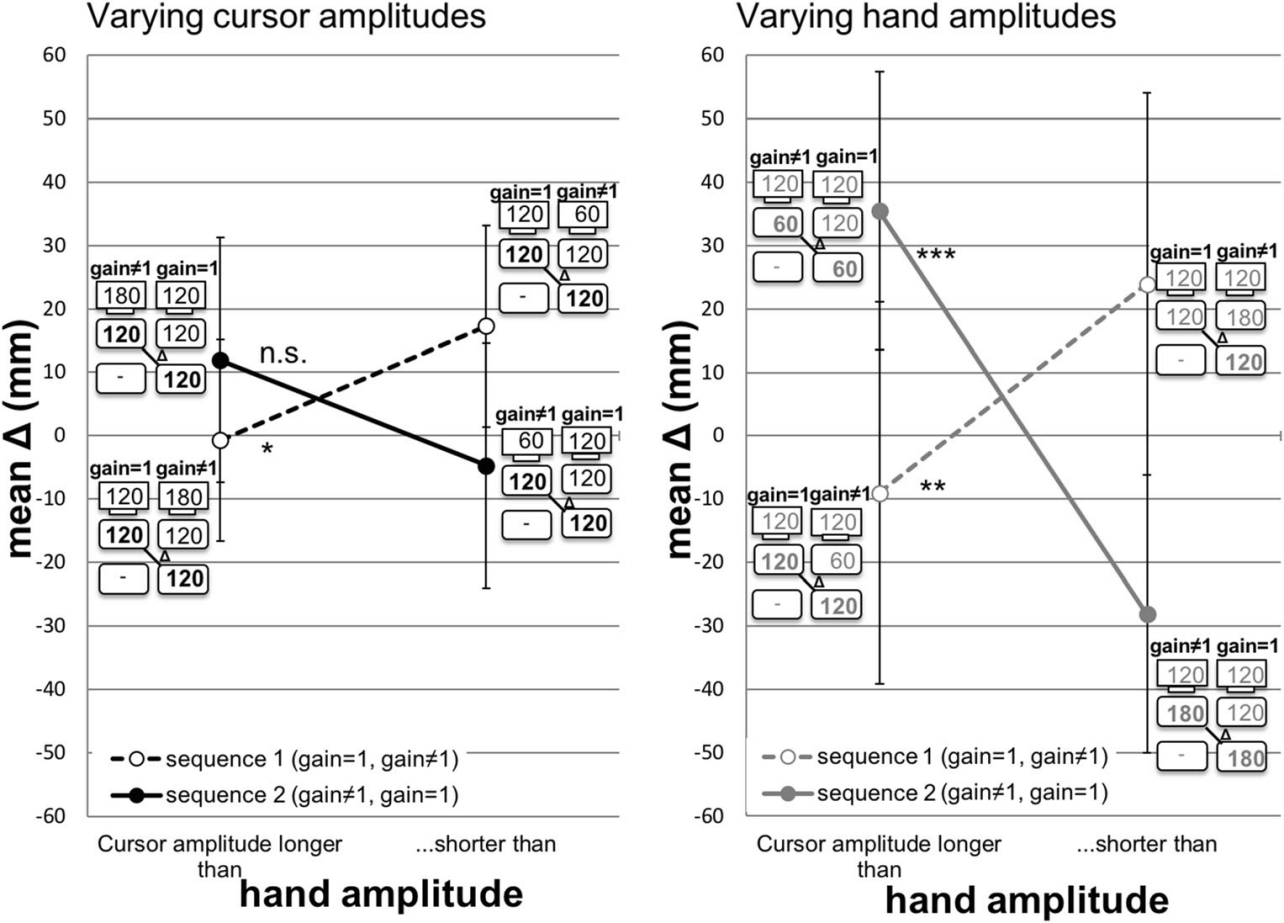
Mean deviation (mm) between the hand amplitude in phase 2 of trial *n* and the hand amplitude in phase 1 of trial *n-1* for sequence 1 (gain=1/gain≠1; *dashed*) and sequence 2 (gain≠1/gain=1; *solid*). Experiment 1 with visual feedback: Varying cursor amplitudes (**left**) and varying hand amplitudes (**right***).* Error bars represent the 95% confidence interval (CI) for the paired difference between two means (condition with cursor amplitude longer – condition with cursor amplitude shorter than hand amplitude) (CI_PD_) (cf., Pfister & Janczyk, 2013). Paired sample *t*-tests: *** *p* = .001, ** *p* < .01, * *p* < .05, *ns p* > .05

Figure 3 depicts the mean deviation between the hand amplitude in phase 2 of trial *n* and the hand amplitude in phase 1 of trial *n-1* for sequence 1 (dashed) and sequence 2 (solid).

#### Varying hand amplitude (Fig. 3, right)

In sequence 1, the gain=1 trial was presented first and precedes the gain ≠1 trial. When the cursor amplitude was longer than the hand amplitude, participants undershot -9.1 mm. When the cursor amplitude was shorter than the hand amplitude, participants overshot 23.9 mm. The difference between over- and undershoot was significant [23.9 vs. -9.1 mm, *F*(1,11) = 10.95, *p* = .007, *η*^2^ = .449]. In sequence 2, the gain=1 trial was presented second, and followed the gain≠1 trial. When the cursor amplitude was longer than the hand amplitude, participants overshot 35.6 mm. When the cursor amplitude was shorter than the hand amplitude, participants undershot -28.2 mm. The difference between over- and undershoot was significant [35.6 vs. -28.2 mm, *F*(1,11) = 21.61, *p* = .001, *η*^2^ = .663]. Both overshoots differed significantly from zero, undershoots only in sequence 2 (Table 1).

**Table 1:** T-test results comparing mean deviation (mm) versus 0 for varying hand amplitudes

	*M*	*SD*	*t*(11)	*p*	95% CI		Cohen's *d*
					Lower limit	Upper limit	
Sequence 1							
Undershoot	-9.1	20.0	-1.57	.145	-21.80	3.66	0.45
Overshoot	23.9	33.0	2.51	.029	2.97	44.88	0.73
Sequence 2							
Undershoot	-28.2	26.5	-3.69	.004	-45.01	-11.35	0.77
Overshoot	35.6	34.7	3.54	.005	13.47	57.62	1.02

*Notes*. *CI* confidence interval, sequence 1 (gain=1/gain≠1), sequence 2 (gain≠1/gain=1)

#### Varying cursor amplitude (Fig. 3, left)

In sequence 1, when the cursor amplitude was longer than the hand amplitude, participants undershot -0.7 mm. When the cursor amplitude was shorter than the hand amplitude, participants overshot 17.3 mm. The difference between over- and undershoot was significant [-0.7 vs. 17.3 mm, *F*(1,11) = 6.26, p = .029, *η*^2^ = .363]. In sequence 2, when the cursor amplitude was longer than the hand amplitude, participants overshot 11.9 mm. When the cursor amplitude was shorter than the hand amplitude, participants undershot -4.7 mm. The difference between over- and undershoot was marginally significant [11.9 vs. -4.7 mm, *F*(1,11) = 3.60, p = .084, *η*^2^ = .247]. Over- and undershoots did not differ significantly from zero (Table 2).

**Table 2:** T-test results comparing mean deviation (mm) versus 0 for varying cursor amplitudes

	*M*	*SD*	*t*(11)	*p*	95% CI		Cohen's *d*
					Lower limit	Upper limit	
Sequence 1							
Undershoot	-0.7	24.6	-0.10	.921	-16.35	14.91	0.03
Overshoot	17.3	27.9	2.16	.054	-0.36	35.04	0.62
Sequence 2							
Undershoot	-4.7	21.8	0.75	.468	-18.55	9.11	0.22
Overshoot	11.9	30.9	1.34	.208	-7.68	31.54	0.39

*Notes*. *CI* confidence interval, sequence 1 (gain=1/gain≠1), sequence 2 (gain≠1/gain=1)

The significant two-way interaction between the factors sequence and gain shows the difference between replication performance in sequence 1 (Fig. 3, dashed) compared to sequence 2 (Fig. 3, solid). In sequence 1, the average difference between over- and undershoot was 25.5 mm (20.6 vs. -4.9 mm). In sequence 2, the average difference between over- and undershoot was 40.2 mm (23.7 vs. -16.5 mm). Results confirm that replication performance is less precise in sequence 2.

The three-way interaction of sequence, constancy, and gain (Fig. 3, left and right) reveals that the differences between over- and undershoots are larger in the condition with varying hand amplitudes (Fig. 3, right) than with varying cursor amplitudes (Fig. 3, left). In sequence 1, differences between over- and undershoots occurred in all trials (Fig. 3, right and left, dashed). In sequence 2, differences between over- and undershoots occurred only in trials with varying hand amplitudes (Fig. 3, right, solid), but not in trials with varying cursor amplitudes (Fig. 3, left, solid). Furthermore, the pattern of over- and undershoots was opposite when comparing deviations in sequence 1 (Fig. 3, dashed) with deviations in sequence 2 (Fig. 3, solid).

In sequence 1, we investigated perception-action interaction that might emerge from the temporal overlap of event files. In case of perception-action interaction, a *contrast effect* should appear: When in phase 1 of trial *n* the cursor amplitude was longer than the hand amplitude, participants undershoot. When in phase 1 of trial *n* the cursor amplitude was shorter than the hand amplitude, participants overshoot. The results show significant differences between under- and overshoots for varying cursor amplitudes and varying hand amplitudes (Fig. 3, dashed). The under- and overshoots follow the pattern of a *contrast effect*. Although under- and overshoots differed significantly, only one out of four deviations differed significantly from zero. According to our definition of after-effects, both differences need to be significant. For varying hand amplitudes, an after-effect (overshoot) occurred and it differed significantly from the undershoot. Our data partially confirmed Hypothesis 1 for varying hand amplitudes, but not for varying cursor amplitudes.

In sequence 2, we investigate after-effects and its temporal persistence within a distinct event file. Comparable to Ladwig et al. (2012, 2013, Exp.1), we assumed an *assimilation effect* when the short-term binding of sensory and motor codes persisted over time: When in phase 1 of trial *n-1* the cursor amplitude was longer than the hand amplitude, participants overshoot. And the other way around, when in phase 1 of trial *n-1* the cursor amplitude was shorter than the hand amplitude, participants undershoot. Here we look at rather contradictory findings (Fig. 3, solid). On the one hand, the difference between over- and undershoots is significant for varying hand amplitudes. The over- and undershoots follow the pattern of an *assimilation effect*, and they differ significantly from zero. For varying cursor amplitudes, we do not find any significant results. Hypothesis 2 was confirmed for varying hand amplitudes, but not for varying cursor amplitudes.

Most surprisingly, none of the hypotheses was confirmed for varying cursor amplitudes. Digging deeper into the findings for varying cursor amplitudes, we found a pattern of over- and undershoots that followed the predicted direction. Statistically, none of the deviations differed significantly from zero. Following our restrictive definition of after-effects, we did not find after-effects. For varying cursor amplitudes, replications were significantly more precise when compared to varying hand amplitudes. A reason for this could be that participants were able to adjust their internal model (e.g., Wolpert et al., 2003; Wolpert & Flanagan, 2001). Former studies using the *n* replication task (Ladwig et al., 2012, 2013, Exp. 1) support this interpretation. They found significant but smaller after-effects for varying cursor amplitudes than for varying hand amplitudes. That means after-effects significantly appeared although the hand amplitude of 120 mm remained constant across phases 1 and 2, and replications could have been performed without any corrections of the previously used motor program (Wolpert & Flanagan, 2001). The finding that visual and haptic information from phase 1 still affected replications speaks in favor of the interaction between perception and action (Hommel et al., 2001). In the *n-1* replication task, participants performed the same hand amplitude of 120 mm in phase 1 of trial *n-1*, and in phases 1 and 2 of trial *n* (Fig. 3, left). Since adjustment can appear either in situations of a gain change between trials (e.g., changing from gain≠1 to gain=1, cf. Rieger et al., 2005; Wendker et al., 2014) or as a refinement within a trial, both led to more precise replications.

For varying hand amplitudes, a different pattern of results appeared. In all but one condition, deviations significantly differed from zero. That means after-effects occurred, and the differences between over- and undershoots were significant as well. Although the pattern of results (partially) confirmed Hypotheses 1 and 2, there could be an additional or alternative explanation for the significant differences between over- and undershoots: In judgment tasks, participants are not inclined to use the full range of possible responses (cf., Teghtsoonian & Teghtsoonian, 1978). For instance, when the hand amplitude in phase 1 of trial *n-1* was 60 mm, and 120 mm in phase 1 of trial *n*, participants overshot by 35.5 mm. Replicated hand amplitude was on average 95.5 mm. When the hand amplitude in phase 1 of trial *n-1* was 120 mm, and 180 mm in phase 1 of trial *n*, participants overshot by 23.9 mm (mean replicated hand amplitude = 143.9 mm). For the other deviations, the same systematic could be applied. Considering that our participants did not use the full range of possible responses in the *n-1* replication task, it remains unclear at this point whether and how this contributed to our results.

## Experiment 2

In order to clarify whether participants did or did not use the full range of possible amplitudes in the *n-1* replication task, we conducted a second experiment. In Experiment 2, we varied the hand amplitude in phase 1 to the same amount as in Experiment 1, but we did not present any stimuli or cursor movement on the display. The withdrawal of visual feedback allowed us to measure the accuracy in motor responses (without the impact of visual feedback), and to determine the range of amplitudes participants used in the *n-1* replication task. There are three possible outcomes when comparing the results for varying hand amplitudes with (Exp. 1) and without visual feedback (Exp. 2): First, if results of Experiment 1 fully reflect that participants did not use the full range of possible amplitudes in the *n-1* replication task, then over- and undershoots should be the same in both experiments (Hypothesis 1a). Second, results of Experiment 1 partially reflect that participants did not use the full range of possible amplitudes in the *n-1* replication task, so that this additively contributed to the observed *assimilation/contrast effect*. In this case, we expect significant but smaller over- and undershoots in Experiment 2 compared to Experiment 1 (Hypothesis 1b). Reason for this are provided by the study by Wendker et al. (2014), who found such additive effects in a *n* replication task. In the condition with visual feedback, task and stimuli were presented on a display, and cursor and hand amplitude either corresponded or did not correspond. In the condition without visual feedback, two plastic barriers restricted the hand amplitude by 25, 50, or 75 mm, respectively. In phase 1, participants moved a pen on a covered tablet from one barrier to the other barrier. In phase 2, participants replicated the formerly performed hand amplitude of 25, 50, or 75 mm. In the condition with visual feedback in phase 1, deviations were larger than in the condition without visual feedback. Nevertheless, when no visual feedback was provided, replications deviated in the same direction as with visual feedback. This underlined the notion that additive factors contributed to the observed pattern of over- and undershoots. Third, in case participants did use the full range of possible amplitudes in the *n-1* replication task, replications in Experiment 2 should be very precise and without any systematic over- and undershoots. Consequently, the over- and undershoots observed in Experiment 1 then fully reflect an *assimilation/contrast effect* (Hypothesis 1c).

### Method

#### Participants

Twelve students (11 female) of the RWTH Aachen University, aged from 20 to 36 years (M= 23.5; SD= 4.9), volunteered. Before taking part in the study, all participants gave their informed consent. All but two participants were right-handed. Participants had normal or corrected-to-normal vision, and received credit for a course or were rewarded for their efforts with 7.50 €.

#### Apparatus, task, and stimuli

The apparatus, task, and stimuli were the same as described in Experiment 1 except for the following changes. We did not provide any visual feedback on the display in phase 1. Beside the same apparatus as in Experiment 1, we placed a perforated plastic plate (size 255 × 255 mm) on the experimenter’s side of the cutout groove. A second experimenter sat opposite the participant and adjusted two plastic blocks (95 × 15 × 9 mm) to the plate. Both blocks served as barriers and limited the distance of hand amplitude in phase 1.

At the beginning of each trial, the second experimenter placed both plastic blocks on the plate 180, 120, or 60 mm apart. In phase 1, participants moved the pen leftward along the groove from the start block to the end block. The second experimenter removed the start block during the participants’ movement. When the participants had reached the end block, phase 2 started. Participants had to reverse the movement direction and to move the pen rightward. Again, phase 2 of trial *n* required to replicate the hand amplitude of phase 1 of trial *n-1* (180, 120, or 60 mm) as accurately as possible.

#### Procedure and design

The procedure and design were the same as described in Experiment 1 except for the following changes. The experiment consisted of a practice block and an experimental block. Before the practice block and experiment started, participants were instructed to produce continuous and smooth forth and back movements with the pen without interrupting. They were further instructed to monitor the hand amplitude carefully since they had to replicate this hand amplitude in the subsequent trial in phase 2 as accurately as possible. At the beginning of each trial, a second experimenter positioned the start block next to the pen and the second (end) block at a distance of 180, 120, or 60 mm. A trial started with a first click of the pen’s button, and participants moved the pen to the opposite block, while the second experimenter removed the start block. When the pen had reached the opposite block, participants pressed the pen’s button a second time. Participants started to replicate the hand amplitude of trial *n-1* by reversing the movement direction with the pen. When it was thought they had replicated the required distance (i.e., hand amplitude of phase 1 of the previous trial), they finally pressed the pen’s button again to terminate the trial. The second experimenter presented the next trial. The experiment lasted about 45 min.

The practice block consisted of four trials with hand amplitude ≠120 and three trials with hand amplitude =120 (in total seven trials). In trials with hand amplitude ≠120 trials, the hand amplitude was 60 or 180 mm in phase 1 (two repetitions each), and in trials with hand amplitude =120, the hand amplitude was 120 mm (three repetitions). The presentation order of trials with hand amplitude ≠120 and =120 was fixed within the practice block. The practice block started and ended with a trial with hand amplitude =120. Practice trials were presented in advance of the experimental block in order to familiarize participants with the task.

The experimental block consisted of 24 trials with hand amplitude ≠120 and 25 trials with hand amplitude =120 (in total 49 trials). The hand amplitude in trials with hand amplitude ≠120 was 60 or 180 mm in phase 1 (12 repetitions each). In trials with hand amplitude =120, the hand amplitude was 120 mm (25 repetitions). The experimental block always started and ended with a trial with hand amplitude =120. Four different sequences arranged the presentation order of trials with hand amplitude ≠120 and =120: In sequence 1 (eight repetitions per amplitude ≠120 condition 60, and 180 mm), the trial with hand amplitude =120 is presented first, and precedes the trial with hand amplitude ≠120. In sequence 2 (eight repetitions per amplitude ≠120 condition), the trial with hand amplitude =120 is presented second and follows a trial with hand amplitude ≠120. In sequence 3 (eight repetitions), a trial with hand amplitude ≠120 preceded a trial with hand amplitude ≠120. In sequence 4 (eight repetitions) a trial with hand amplitude =120 preceded a trial with hand amplitude =120. Sequences 3 and 4 were presented to avoid sequence-learning effects. The order of sequences was pseudo-random.

Similar to Experiment 1, the first experimental trials (=120) were excluded before error analysis (2.04% of all trials). The error treatment followed the same criteria as described in Experiment 1. In total, the error rate was 12.15%.

To determine the sample size for sufficient statistical power, the same considerations as in Experiment 1 applied to Experiment 2. Data were analyzed using a 2 × 2 × 2 mixed ANOVA with repeated measurement with the between-subject factor visual feedback (with (Exp. 1) vs. without (Exp. 2)) and the within-subject factors sequence (1 (=120/≠120) vs. 2 (≠120/=120)), and amplitude (60 vs. 180 mm). We used *t*-tests to analyze whether mean deviations differed significantly from zero, and calculated Cohen’s *d* for the effect size. As the dependent variable, the mean deviation (in mm; see Fig. 1, dashed arrow = ∆) between the hand amplitude in phase 2 of trial *n* and the hand amplitude in phase 1 of trial *n-1* was calculated for error-free trials. Positive values of mean deviations represent an overshoot in phase 2, negative values of mean deviations represent an undershoot in phase 2.

### Results and discussion

The 2 × 2 × 2 mixed ANOVA revealed a significant main effect of the factor amplitude [*F*(1,22) = 6.52; *p* = .018, *η*^2^ = .22], a significant interaction of sequence and amplitude [*F*(1,22) = 16.49; *p* = .001, *η*^2^ = .42], a significant interaction of visual feedback and amplitude [*F*(1,22) = 6.53; *p* = .018, *η*^2^ = .22], and a significant interaction of visual feedback, sequence, and amplitude [*F*(1,22) = 13.44; *p* = .001, *η*^2^ = .37]. Other main effects and interaction did not reach significance (factor sequence [*F*(1,22) = 0.38; *p* = .539, *η*^2^ = .01], factor visual feedback [*F*(1,22) = 0.34; *p* = .561, *η*^2^ = .01], interaction of sequence and visual feedback [*F*(1,22) = 3.48; *p* =.075, *η*^2^ = .13]).

**Fig. 4:**
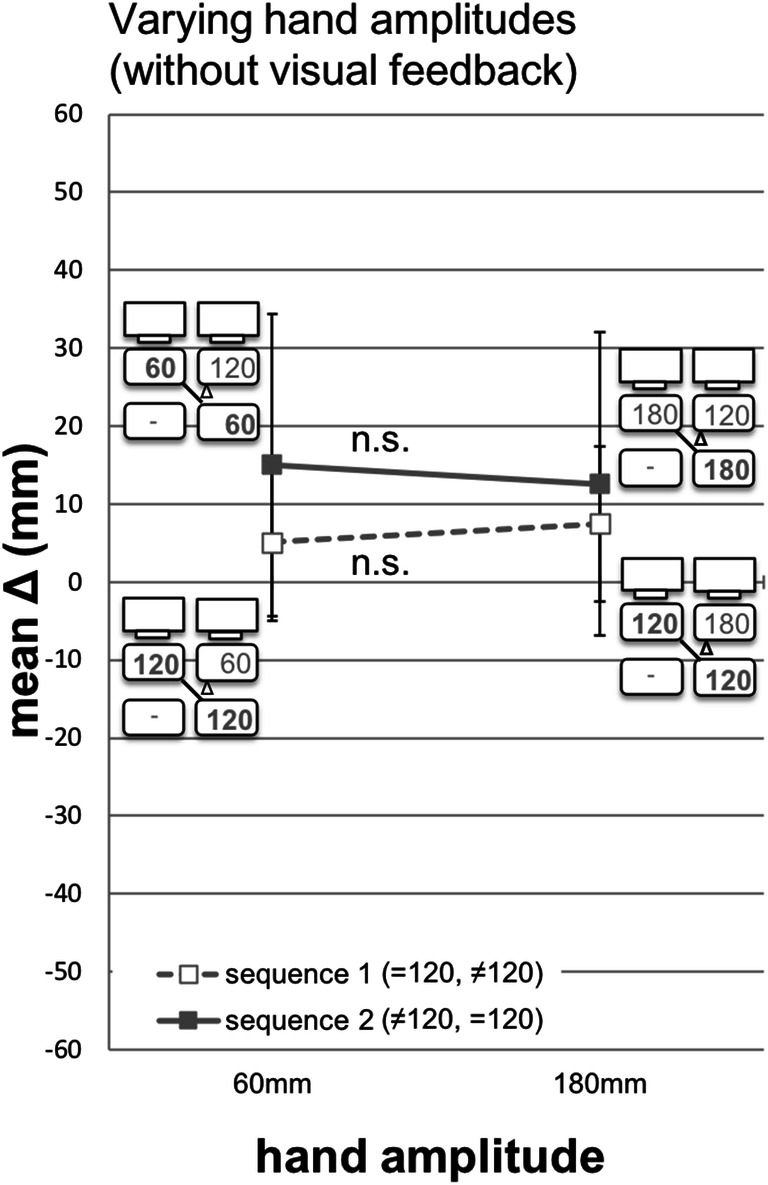
Mean deviation (mm) between the hand amplitude in phase 2 of trial *n* and the hand amplitude in phase 1 of trial *n-1* for sequence 1 (=120/≠120; *dashed*) and sequence 2 (≠120/=120; *solid*). Error bars represent the 95% confidence interval (CI) for the paired difference between two means (condition with hand amplitude =120 – condition with hand amplitude ≠120) (CI_PD_) (cf., Pfister & Janczyk, 2013). Paired sample *t*-tests: *** *p* = .001, ** *p* < .01, * *p* < .05, *ns p* > .05

Figure 4 depicts the mean deviation between the hand amplitude in phase 2 of trial *n* and the hand amplitude in phase 1 of trial *n-1* for sequence 1 (=120/≠120; *dashed*) and sequence 2 (≠120/=120; *solid*). In the following, the three-way interaction of visual feedback, sequence, and amplitude (Fig. 3, right, and Fig. 4) are described in detail.

In sequence 1, the trial with gain=1 (with visual feedback) and with the hand amplitude =120 (without visual feedback) was presented first, and preceded the trial with gain≠1 and with amplitude ≠120*.* With visual feedback, the difference between over- and undershoots was significant. Without visual feedback, participants overshot 5.1 mm when the hand amplitude in phase 1 was 60 mm. When the hand amplitude in phase 1 was 180 mm, participants overshot 7.6 mm. The overshoots did not differ significantly from each other [5.1 vs. 7.6 mm, *F*(1,11) = 0.299*, p* = .595, *η*^2^ = .027].

In sequence 2, the trial with gain=1 and with the hand amplitude =120 was presented second, and followed a trial with gain≠1 (with visual feedback) and with amplitude ≠120 (without visual feedback)*.* With visual feedback, the difference between over- and undershoots was significant. Without visual feedback, participants overshot 15.1 mm when the hand amplitude in phase 1 was 60 mm. This overshoot differed significantly from zero (Table 3). When the hand amplitude in phase 1 was 180 mm, participants overshot 12.6 mm. The overshoots did not differ significantly from each other [15.1 vs. 12.6 mm, *F*(1,11) *=* 0.078, *p* = .785, *η*^2^ = .007].

**Table 3:** T-test results comparing mean deviation (mm) versus 0 for varying hand amplitudes without visual feedback

Mean deviation when hand amplitude is	*M*	*SD*	*t*(11)	*p*	95% CI		Cohen's *d*
					Lower limit	Upper limit	
Sequence 1							
60 mm	5.1	27.3	0.65	.532	-12.25	22.42	0.19
180 mm	7.6	21.4	1.23	.246	-6.03	21.15	0.35
Sequence 2							
60 mm	15.1	14.3	3.65	.004	5.97	24.14	1.05
180 mm	12.6	27.5	1.59	.141	-4.90	30.09	0.46

*Notes*. *CI* confidence interval, sequence 1 (=120, ≠120), sequence 2 (≠120,=120)

Systematic over- and undershoots were present with visual feedback, and all but one deviation differed significantly from zero (Table 1; sequence 1 – undershoot). Without visual feedback, deviations did not vary systematically across conditions, and they did not differ significantly from zero, except in one condition (Table 3; sequence 2 – 60 mm).

In Experiment 2, we do not find any significant effect. Replication performance is quite precise without visual feedback, and it does not differ between conditions. There is a slight general inaccuracy in all replications. However, we did not find any systematic deviation indicating that participants did not use the full range of possible amplitudes in the *n-1* replication task. Consequently, our Hypotheses 1a and 1b could not be confirmed. The results are in favor of Hypothesis 1c. The pattern of systematic and significant over- and undershoots was present in Experiment 1, when visual feedback was provided that did or did not correspond to haptic feedback. In Experiment 2, when participants did not receive any visual feedback, replications were quite precise, and did not vary systematically or significantly. This confirms Hypothesis 1c. Consequently, the observed over- and undershoots in Experiment 1 can be interpreted as a *contrast effect* (sequence 1) and *assimilation effect* (sequence 2).

## General discussion and conclusions

In two experiments, we investigated the temporal persistence of after-effects in a distinct event file, and after-effects in temporally overlapping event files in a sensorimotor transformation task. In the *n-1* replication task, each trial consisted of two phases. In phase 1 of trial *n,* participants moved a pen on a covered digitizer tablet while a gain varied the relation between hand amplitude and cursor amplitude. In phase 2 of trial *n*, participants replicated the hand amplitude of phase 1 of trial *n-1* without visual feedback. Two sequences varied the order of gain=1 trials and gain≠1 trials to disentangle the temporal persistence of after-effects in a distinct event file, and after-effects in temporally overlapping event files.

First, we tried to capture the modulating influence of temporally overlapping event files on replication performance. In sequence 1, a gain=1 in trial *n-1* (relation between cursor and hand amplitude is 120:120) was presented first, and preceded a gain≠1 in trial *n* (e.g., 60:120). We assumed that sensory and motor codes of event file 1 (gain=1) do not interact, but motor codes of event file 1 might be affected by the temporally overlapping sensory codes of event file 2 (gain≠1). If temporally overlapping event files modulate perception-action interaction, we should observe a *contrast effect* (Hypothesis 1). Adaptation or adjustments of the internal model (Wolpert & Flanagan, 2001) would predict the same outcome. The significantly different under- and overshoots followed the pattern of a *contrast effect*. Although under- and overshoots differed significantly, only one out of four deviations differed significantly from zero. According to our definition of after-effects, both differences need to be significant. For varying hand amplitudes, an after-effect (overshoot) occurred and it differed significantly from the undershoot. Our data in Experiment 1 partially confirmed Hypothesis 1 for varying hand amplitudes, but not for varying cursor amplitudes.

Second, we investigated the short-term binding of sensory and motor codes within a distinct event file (Hommel et al., 2001). In line with TEC (Hommel et al., 2001), perception is likely to interact and to affect action. In sequence 2, the gain=1 trial (120:120) was presented second, and followed the gain≠1 trial (e.g., 120:60). We assumed that sensory and motor codes of event file 1 (gain≠1) interact. The temporally overlapping sensory codes of event file 2 (gain=1) should not affect motor codes of event file 1, since sensory codes of event file 2 did not cause any conflict. An *assimilation effect* was expected (Hypothesis 2). And this is exactly what we found for varying hand amplitudes: When in phase 1 of trial *n-1* the cursor amplitude was longer than the hand amplitude, participants overshot. When in phase 1 of trial *n-1* the cursor amplitude was shorter than the hand amplitude, participants undershot. The *assimilation effect* for varying hand amplitudes was statistically confirmed. For varying cursor amplitudes, we did not find any significant results. To sum up, for varying hand amplitude our data confirmed Hypothesis 2. For varying cursor amplitudes, Hypothesis 2 was not confirmed. Ladwig et al. (2012, 2013) already reported for the *n* replication task that sensory information (phase 1) and motor response (phase 2) assimilated. The *assimilation effect* was significantly smaller for varying cursor amplitudes than for varying hand amplitudes. Contrary to that, we did not find any statistically significant after-effects for varying cursor amplitudes at all.

This will be discussed in more detail. When cursor amplitudes varied, hand amplitudes remained constant across trials. In the present experiment, it seemed that participants adjusted their internal model and motor execution became more precise (cf., Wolpert & Flanagan, 2001). This reduced or even eliminated after-effects in such a way that they were not statistically significant. The studies by Ladwig et al. (2012, 2013) did not control gain sequences as we did. Wendker and colleagues re-analyzed their data for gain repetitions and gain changes between two consecutive trials, and found a reduction of after-effects when the hand amplitude remained constant across two consecutive trials. This underlines the fact that adjustments between trials (e.g., changing from gain≠1 to gain=1, cf. Rieger et al., 2005; Wendker et al., 2014) or refinements within a trial occurred very fast, and both led to a more precise performance. Furthermore, top-down processes altering short-term binding of sensory and motor codes could also be responsible for our ambiguous findings of after-effects. The cognitive load to perform the *n-1* replication task is obviously very high, as motor information from trial *n-1* has to be stored and retrieved for replication in trial *n*. According to Westbrook and Braver (2015), the concept of cognitive effort describes the intensity of engagement in a task. We can only speculate at this point whether cognitive effort and/or focused attention on the task at hand attenuated short-term binding, and consequently reduced or eliminated after-effects at all (e.g., Sack & Sutter, 2017).

When hand amplitudes varied, cursor amplitudes remained constant across trials. We found a *contrast effect* (sequence 1) and an *assimilation effect* (sequence 2) in Experiment 1. An alternative explanation for our observed pattern of over- and undershoots could be that in judgment tasks, participants are not inclined to use the full range of possible responses (cf., Teghtsoonian & Teghtsoonian, 1978). In a second experiment, we clarified whether participants did or did not use the full range of possible amplitudes in the *n-1* replication task. Participants performed the same replication task as in Experiment 1 but did not receive any visual feedback in phases 1 and 2. If participants did not use the full range of possible amplitudes over- and undershoots should be the same in Experiment 1 and 2 (Hypothesis 1a). If this additively contributed to the observed after-effects in Experiment 1, then over- and undershoots should be larger in Experiment 1 compared to Experiment 2 (Hypothesis 1b). In case, participants did use the full range of possible amplitudes replications in Experiment 2 should be very precise, without any significant or systematic deviation (Hypothesis 1c). Consequently, the observed over- and undershoots in Experiment 1 fully reflect an *assimilation/contrast effect*. Wendker et al. (2014) introduced amplitude replications without visual feedback in their study with the *n* replication task. In two conditions, they presented transformed visual feedback in phase 1 or did not present any visual feedback, respectively. They found, over- and undershoots varied to a greater extend in the condition with transformed visual feedback than without visual feedback. The additional amount of over- and undershoots in the condition with transformed visual feedback were interpreted as *assimilation effect*. We adapted their approach to our *n-1* replication task. Depending on the amount of after-effects in Experiment 1, we assumed equal, smaller, or no systematic over- and undershoots in Experiment 2 without visual feedback (Hypotheses 1a, 1b, and 1c, respectively). Most interestingly, we did not find any systematic over- and undershoots in Experiment 2, and three out of four deviations did not differ significantly from zero. This result confirms Hypothesis 1c, so that over- and undershoots observed in Experiment 1 indeed reflect perception-action perception in terms of *assimilation/contrast effects*.

For varying hand amplitudes, the findings provide evidence for the temporal persistence of short-term bindings of sensory and motor codes within a distinct event file (Hommel et al., 2001). In our case, the binding persisted from phase 1 of trial *n-1* to phase 2 in trial *n*. This corresponds to a temporal delay of approximately 10–20 s. Woods, O’Modhrain, and Newell (2004) investigated the temporal delay in cross-modal object recognition. Participants had to match two L-shaped objects of different sizes. One of the objects was presented visually, the other one was presented haptically, or the other way around. There was a time delay of 0, 15, or 30 s between the first and the second stimuli. As a result, performance decreased with a time delay of 30 s, but not with a shorter delay. This confirms that sensory information can be stored and integrated within a time interval of at least 15 s. For varying hand amplitudes, we furthermore found after-effects from temporally overlapping event files. We replicated the *contrast effect* by Schubö et al. (2001) in our *n-1* replication task.

Future studies should be conducted to clarify the absence of after-effects for varying cursor amplitudes. The first step would be to adapt the experimental design of controlling gain order to the *n* replication task, and investigate after-effects in the same sequences as defined in our Experiment 1. So far, all experiments using the *n* replication task presented gains randomly (Ladwig et al., 2012, 2013; Perrotin & d’Alessandro, 2016; Sack & Sutter, 2017; Wenker et al., 2014), and results are not completely comparable to the present findings. In a second step, further studies could shed light on the top-down influence we speculated about in the discussion above. If high cognitive load is responsible for the attenuation of after-effects in the *n-1* replication task, then similar results should be found with increased cognitive load in the *n* replication task.

To sum up, in the *n-1* replication task, we investigated the temporal persistence of after-effects in a distinct event file, and after-effects in temporally overlapping event files in Experiment 1. For varying cursor amplitudes, performance was more precise than for varying hand amplitude, and we did not observe any predicted effect. For varying hand amplitudes, we found both effects. Short-term binding of sensory and motor codes persisted over time and triggered an *assimilation effect*. The interaction between temporally overlapping event files resulted in a *contrast effect*. In Experiment 2, we ruled out that participants did not use the full range of possible amplitudes in the *n-1* replication task. Consequently, our observed pattern of over- and undershoots in Experiment 1 can be interpreted as an *assimilation/contrast effect*. The results extend the current view on temporal stability of short-term bindings in sensorimotor tasks: Bindings temporally persist up to approximately 20 s. The same time delay applies to the temporal persistence of multisensory integration in the combined concept (Woods et al., 2004).
